# A Case Study of a Retracted Systematic Review on Interactive Health Communication Applications: Impact on Media, Scientists, and Patients

**DOI:** 10.2196/jmir.7.2.e18

**Published:** 2005-06-30

**Authors:** Roy Rada

**Affiliations:** ^1^Department of Information SystemsUniversity of MarylandBaltimoreMDUSA

**Keywords:** Retraction of publication, online systems, mass media, patients, medical errors, editorial policies

## Abstract

**Background:**

In October 2004, a flawed systematic review entitled “Interactive Health Communication Applications for People with Chronic Disease” was published in the Cochrane Library, accompanied by several press releases in which authors warned the public of the negative health consequences of interactive health communication applications, including the Internet. Within days of the review's publication, scientists identified major coding errors and other methodological problems that invalidated the principal conclusions of the study and led to a retraction. While the original study results and their negative conclusions were widely publicized in the media, the retraction seemed to go unnoticed.

**Objective:**

This paper aims to document an unprecedented case of misinformation from a Cochrane review and its impact on media, scientists, and patients. As well, it aims to identify the generic factors leading to the incident and suggest remedies.

**Methods:**

This was a qualitative study of the events leading to the retraction of the publication and of the reactions from media, scientists, and patients. This includes a review and content analysis of academic and mass media articles responding to the publication and retraction. Mass media articles were retrieved in May 2005 from LexisNexis Academic and Google and were classified and tallied. The extended case method is employed, and the analysis is also applied to comparable publishing events.

**Results:**

A search on LexisNexis Academic database with the query “Elizabeth Murray AND health” for the period of June 2004 to May 2005 revealed a total of 15 press reports, of which only 1 addressed the retraction. Google was searched for references to the review, and the first 200 retrieved hits were analyzed. Of these, 170 pages were not related to the review. Of the remaining 30 pages, 23 (77%) were reports about the original publication that did not mention the retraction, 1 (3%) was a bibliography not mentioning the retraction, and 6 (20%) addressed the retraction, of which only 1 was a non-Cochrane–related source.

**Conclusions:**

Analyzed retrievals showed that the mass media gave more coverage to the Cochrane review than to the retraction or to a related systematic review with a similar scope but a different conclusion. Questionable results were prematurely disseminated, oversimplified, and sensationalized, while the retraction was hardly noticed by the public. Open commentary by scientists and patients helped to rapidly identify the errors but did not prevent or correct the dissemination of misinformation.

## Introduction

### Publication of the Review

On October 18, 2004, the Cochrane Collaboration, a organization which produces and disseminates systematic reviews of health care interventions [1], published a review entitled “Interactive Health Communication Applications for People with Chronic Disease” [2], which from this point on will be referred to as the “IHCA review.” The IHCA review was edited by the Cochrane Consumers and Communication Review Group [3]. Those who prepare reviews volunteer to work in one of many Collaborative Review Groups, with editorial teams overseeing the preparation and maintenance of the reviews.

Interactive health communication applications (IHCAs) were defined in the IHCA review as “computer-based, usually Web-based, health information packages for patients that combine information with social-, decision-, or ‘behavior change'-support” [2]. The results of the IHCA review showed that IHCAs had a positive effect on knowledge and on social support, no effect on behavioral outcomes, and a negative effect on clinical outcomes.

The principal conclusion of the review was “consumers whose primary aim is to achieve optimal clinical outcomes should not use an IHCA” [2]. This conclusion was the focus of a press release which the mass media widely circulated (as will be documented later). However, only days later, the IHCA review was found to be flawed and was retracted.

### Retractions

The National Library of Medicine (NLM) is a leader in the bibliographic handling of retractions. The Medical Subject Headings (MeSH) contain the concept “retracted publication,” which identifies a citation previously published and now retracted through a formal issuance from the author, publisher, or other authorized agent. In January 2005, the PubMed query “Retracted Publication[Publication Type] AND 1971:2004[edat]” retrieved 619 retracted citations that entered PubMed between 1971 and 2004. Since the query “1971:2004[edat]” retrieves approximately 12.5 million citations, less than 1 in 10000 publications have been retracted.

Friedman [4] studied 60 fraudulent articles by one scientist. Journals in which the scientist had published were notified of the fraud. Only 7 articles were subsequently tagged in PubMed with “Retracted Publication.” The delay between publication of a paper and its retraction often has deleterious effects [5]. Furthermore, journals and institutions are hesitant to issue a statement of errors in published work unless the author of the work confesses to the error, which authors may resist doing because such an admission can be career-damaging.

While very few publications are officially retracted, the concern about factors related to retractions is substantial. The study of retractions itself might be indexed with MeSH concepts such as “scientific misconduct,” although the fraction of retractions that stem from error as opposed to scientific misconduct is not known. The query “Scientific Misconduct[majr] AND 1971:2004[edat]” in PubMed retrieved 1840 citations. This body of literature recommends that medical researchers constructively criticize the research practices of others in their institution to reduce the likelihood of misconduct [6].

The objective of this paper is to document the IHCA review as an event in the history of medical publishing, to identify the factors leading to the publicizing of a retracted publication, and to assess the implications.

## Methods

The objectives of this research called for various study methods. The author employed the following three methods: (1) historical processes of collecting documents about a contemporary event and organizing them thematically; (2) ethnographic processes of author participation in the event, personal communication with other participants in the event, interpretation of communications, and construction of models; (3) content analyses based on bibliographic database and Internet searches, coding of the retrieved documents, and tallying of the code frequencies.

The ethnographic method employs the extended case method, and the extended case method applies reflexive science to ethnography. Buroway describes reflexive science as follows: “Reflexive science starts out from dialogue, virtual or real, between observer and participants, embeds such dialogue within a second dialogue between local processes and extralocal forces that in turn can only be comprehended through a third, expanding dialogue of theory with itself” [7].

Various database and Internet searches were employed to study the impact of the review and to quantify the difference between mass media coverage of the original publication and its retraction. LexisNexis Academic databases of health news and general news were searched, as was Google. The queries were designed in an iterative process that began with keywords from the question to be addressed but refined the query based on study of the query retrieval results. The retrieved results were coded, and the coding language was also developed in an iterative process. First, the obvious codes “about the review” and “about the retraction” were introduced. Each retrieved document was classified into a single code by the author. If the retrieved document was not appropriately described by an existing code, then the coding language was augmented. The Web of Science was also queried to identify academic citations, but no citations were identified (data not shown). Most database and Internet searches were conducted in May 2005.

To better understand how special the publicity accorded the IHCA review was, this study was extended to three other publications: 2 of these were retracted publications tagged as “Retracted Publication” (1 Cochrane review, but not eHealth related, and 1 non-Cochrane review, but eHealth related), and 1 was a meta-analysis with a scope similar to that of the IHCA review. These 3 reports were identified through PubMed searches.

## Results

The following qualitative results on the impact of the IHCA review are organized into three main sections: scientist reaction, mass media reaction, and patient reaction.

The section on scientist reaction considers Cochrane reviewers' reactions and how eHealth scientists responded to the IHCA review in the comment section of the Cochrane database. The mass media section provides the Cochrane retraction and then explores, via LexisNexis and Google results, the reaction of the mass media to the IHCA review. The patient reaction section shares dialogue from patient-patient online discussions that reveals the reactions of patients to the IHCA review.

### Scientist Reaction

The Cochrane Collaboration allows anyone to submit comments to the published reviews. Two scientists' comments on the IHCA review appeared independently on October 28, 2004. Kummervold and Eysenbach criticized the IHCA review for both its protocol and its coding.

Kummervold explained in detail how the coding of the meta-analysis was incorrect: “We can't get the numbers to add up, it looks like they are reversed in 8 of the 11 studies...” [8]. He delineated the facts and the interpretation for each of the 8 studies at issue; for example, regarding the HbA1c measurement in the Lehmann 2003 paper, he stated that Lehmann reported a reduction in HbA1c of 0.8 for the intervention group, and 0.1 for the control group, which should be interpreted as a positive result for the intervention group. Kummervold added: “We also find it strange that you focus so much on the overall estimates when there is so much heterogeneity in the material. The conclusion seems to be overstated” [8].

Eysenbach had similar comments, stressing that a formal meta-analysis of these heterogeneous studies was problematic, and that the three studies which contributed most to the “negative” result were in fact positive: “Apart from the fact that I do not think that it is legitimate to do a formal meta-analysis using papers measuring totally heterogeneous outcomes with different types of interventions, I also notice that the overall effect estimate is ‘negative' (eg, ‘favoring control') because of three studies…. However, when I read these three studies I cannot find that their result[s] are negative…. If my suspicion is correct, then this is quite a catastrophic error, and quite an embarrassment for Cochrane to let such an error slip through peer-review” [9].

On November 10, 2004, the Cochrane Consumers and Communication Review Group reacted to the discovered errors [10] with a notice that included the following: “The review will be withdrawn as soon as possible…. As the corrections to the review have not been completed yet, it would be premature to announce any reversal of the review's findings at this stage.… The original press releases regarding this review were made not by the Cochrane Collaboration itself but by University College London….”

John Wiley & Sons (the publisher of the Cochrane Database) released to EurekAlert a retraction on December 6, 2004: “The review originally determined that…chronically ill people using interactive programmes had worse clinical outcomes than those who did not. Regrettably, errors in data analysis meant that these outcomes were reported incorrectly.... It is expected that the revised results will be published in April 2005” [11].

The April 2005 edition of the Cochrane Systematic Reviews did not mention the IHCA review. Royle, the chief executive officer of the Cochrane Collaboration, said that further review of the revised report was ongoing and no date could be given as to when the review might be published (personal communication, April 25, 2005).

### Mass Media Reaction

The Cochrane Database of Systematic Reviews is not read by the typical consumer. However, Murray's employer, the University College London (UCL), worked with Murray to widely publicize the result. UCL posted a news bulletin on its website on October 18, 2004 that remained there as of May 25, 2005. The bulletin was titled “Knowledge may be hazardous to web consumers' health” and stated the following: “People who use their computers to find information about their chronic disease often wind up in worse condition than if they had listened to their doctor, according to a UCL review of studies on internet health.… One reason…might be because knowledge-seekers become so steeped in information from the Internet they make treatment choices on their own, contradicting advice from their doctors” [12].

Most significantly, the UCL bulletin was circulated to information intermediaries that are considered the main entrance to the world's mass media, including AlphaGalileo and EurekAlert.

A search on LexisNexis Academic with the query “Elizabeth Murray AND health” for the period June 2004 to May 2005 revealed a total of 15 relevant press reports, in the following categories:

- Medical and Health News: There were 9 publications with titles such as UCL's press release title of “Knowledge may be hazardous to web consumers' health.” The publications appeared in places like *Life Science Weekly*, *Law and Health Weekly*, and *Health and Medicine Week.*
- General News–Major Papers: There were 5 relevant articles, such as one entitled “Why medical advice from the internet can be bad for your health” in the British *The Daily Telegraph* and another entitled “Medical Web sites may be unhealthy places to learn about ills” in the *Omaha World Herald*. Only 1 article was about the retraction, published in the Ottawa Citizen on October 18, 2004.
- Time Incorporated Publications: There was 1 article in the November 1, 2004 issue of *Time* entitled “Click to Get Sick?” [13].

Among the 15 results from the LexisNexis Academic database, only 1 newspaper report, authored by Tom Spears, dealt specifically with the retraction [14]. Spears, in personal communication with this author (November 18, 2005), said, “I was fairly stunned today to learn that it [IHCA review] has been withdrawn; I found out only because I was looking up the study for my daughter, a science student. Now I'm covering the sequel for tomorrow's paper.… I scan EurekAlert faithfully, as many reporters do, and never saw a hint of anything there.”

To further test whether the media emphasized the false negative result but minimally covered the retraction, a content analysis on Google was performed on May 24, 2005. The query was “health AND Cochrane AND Murray AND (interactive OR web OR internet)” for English pages, within the past year. Of the first 200 retrieved hits, 170 pages were not related to the IHCA review. Of the remaining 30 pages, 23 (77%) were reports about the original publication that did not mention the retraction, and an additional page was a bibliography (at a UCL site) that included a citation to the IHCA review, again without mentioning the retraction. All reports (except the bibliography) used a title such as “Click to Get Sick?” and emphasized the negative impact on clinical outcomes of using the Web. The reports came from such reputable sources as the *British Broadcasting Corporation* and *US News and World Report*. In contrast, only 6 pages (20%) addressed the retraction: 2 were the original press releases now marked with “retraction” but still emphasizing in their particulars the negative health impact, 3 were Web pages at Cochrane sites, and 1 was an announcement from *MedicalNews* entitled “Updated press release to October 2004 Cochrane Review.” The latter was the only non-Cochrane–related page primarily addressing the retraction.

The grey literature reported on the mass media. For example, *The Neuroscience for Kids Newsletter* summarized [15] the “Click to Get Sick?” *Time* article by Sanjay Gupta, and a Web archive for patient education at the Samaritan Health Center pointed patients to Gupta's article. This author wrote to Gupta and asked him to write about the retraction, but Gupta did not reply.

NLM indexed the IHCA review and entered the citation for it (including its abstract) in PubMed on October 21, 2004. The “Retracted Publication” tag did not, however, appear in PubMed until March 24, 2005.

### Patient Reaction

Some patients reported the news about the IHCA review to their patient-patient online discussion groups. In a neurology patient discussion group [16], a patient posted the entire BBC news story. Patients responded in two ways. Some rejected the IHCA review result and added strong comments, such as “I have gotten more help and answers for problems from knowledgeable people on this Internet Forum than I have from any of the multitude of doctors I have seen over the last 12 years.” Others accepted the conclusion but insisted that patients could filter bad information from good and benefit in the end from the web. These patients were not aware of the retraction of the IHCA review.

This author reported the *Time* “Click to Get Sick?” article to two head-and-neck cancer patient discussion groups to which he belongs. A day later he reported the retraction from the Cochrane Database. One member of the discussion group replied: “Thanks for the update–the negative findings seemed odd to me when I read it, so I'm glad it's being revised.” This author, in his role as a cancer patient, also formally commented on the IHCA review at the Cochrane Database site [17].

The typical patient with a chronic disease has no formal medical training and is ill prepared to critique a meta-analysis of clinical trials. However, the typical patient is vulnerable to cultural pressures, as they are partially shaped by and reflected in the mass media.

### Comparison With Another Cochrane Retraction

For comparison, a search for further retracted Cochrane reviews using the PubMed query “Cochrane Database Syst Rev[TA] AND Retracted Publication[PT] AND 1971:2005/5/25[edat]” was conducted. One reference, in addition to the IHCA review already discussed, was identified, which was a retracted review by Brewster et al [18] about antihypertensives. The retraction for the Brewster et al review is explained on the Cochrane website as follows: “This systematic review has been withdrawn temporarily because its contents are potentially misleading.”

A search on LexisNexis with the query “Brewster AND antihypertensive” for the period November 2004 to May 2005 retrieved no articles in either the “General News–Major Papers” category or the “Medical and Health News” category.

A search on Google for “Brewster antihypertensive” followed by an examination of the first 100 retrieved pages identified 23 relevant pages, which had a very different content pattern than the hits for the IHCA review. They all contained citations of papers from Brewster et al, who have published elsewhere on the same subject as in their review. The Brewster et al publication attracting the most attention was an article [19] in the *Annals of Internal Medicine* that was not retracted but has the same title as the Cochrane review. Thus, the only other retracted Cochrane review had a very different mass media, scientific, and web impact than the IHCA review.

### Comparison With Other Retracted Articles Related to eHealth

To determine whether other articles on a similar topic to the IHCA review have been retracted, a search was first made for articles on a similar subject that had been MeSH indexed in PubMed. The article by Demiris [20] seemed relevant, and its two MeSH index terms were “Disease Management” and “Internet.” A search on PubMed for “Retracted Publication[PT] AND Disease Management[majr] AND Internet[majr] AND 1995:2005/5/25[edat]” returned no citations. When the search was broadened by removing the term “Disease Management,” 1 retracted reference was retrieved, entitled “The quality of surgical information on the Internet” [21]. As previously described in the Journal of Medical Internet Research, this article was retracted due to a case of cyberplagiarism, with large sections of the paper having been lifted from different websites [22].

A search on LexisNexis Academic with the query “McKinley and surgical and Internet” for the period 1995 to May 2005 revealed no relevant press reports, neither in the “General News–Major Papers” category (three hits were all not relevant to the McKinley article) or in the “Medical and Health News” category.

A search on Google for English pages with the query “McKinley surgical Internet” revealed 96 irrelevant pointers in the first 100 results. Of the remaining 4 relevant hits, 1 was the article about the plagiarism [22], which precipitated the retraction of the McKinley et al manuscript, and 3 were academic references to the McKinley et al article, which did not note it being retracted.

Thus, the only other retraction of a published article appearing in PubMed similar in topic (the Internet) to the IHCA review had a very different pattern of reactions than the IHCA review.

### A Similar Meta-Analysis on eHealth

The IHCA review addressed a topic that the mass media found interesting. Have any other recent publications also been a meta-analysis on the impact of interactive applications on health, and, if yes, what was the mass media reaction? Using the query “Meta-analysis AND Web AND Chronic Illness” in PubMed, we found only 1 citation: Wantland et al [23] did a meta-analysis on Web-based health interventions that was published (in the Journal of Medical Internet Research) about the same time as the IHCA review. The paper concluded that “the effect size comparisons in the use of Web-based interventions compared to non-Web-based interventions showed an improvement in outcomes for individuals using Web-based interventions to achieve the specified knowledge and/or behavior change for the studied outcome variables.”

What has been the impact of the Wantland et al paper and how does that compare to the impact of the IHCA review? The Wantland et al paper was not announced with a press release in EurekAlert. A search on LexisNexis Academic for newspaper articles about the Wantland et al paper retrieves no articles. The queries performed were similar to those performed for the IHCA review and included “Wantland AND health” for 2004 through 2005 in General News/Major Papers.

A search was done on Google for “Wantland health Web” on May 24, 2005. Of the first 200 returns, 182 were not relevant. Of the remaining 18 hits, 15 pages contained academic citations to Wantland et al, 2 announced the appearance of the article, and 1 was a personal blog that commented on the article.

Thus, most of the Google returns that gave Wantland et al citations are academic in character and very different from the mass media coverage afforded the IHCA review.

## Discussion

As shown, the IHCA review provides a perhaps unprecedented case from which lessons should be drawn. Only one other Cochrane review (about antihypertensives) has been retracted, and that one received negligible mass media attention. The only retracted publication in PubMed that is indexed under the MeSH concept of “Internet” (the IHCA review did not have time to get indexed before it was withdrawn) received no newspaper coverage. The paper most similar to the IHCA review in topic and method (the Wantland et al report [23]) received considerable academic attention but no newspaper coverage. In other words, special circumstances must have come together for the IHCA review situation.

This section next presents a framework based on tiers of response. The first tier is medical scientists. The second tier is the mass media spreading medical press releases. The third tier is the patient community reacting to the mass media and the scientists.

### First-Order Problem

In an effort to critique the problem that occurred, one might build on the analysis of misconduct in toxicology by Purchase. Purchase [24] identified four roots of misconduct:

- Intention of the work
- Conduct of the studies
- Design and interpretation of studies
- Bias from conflict of interest

In the case of the IHCA review, the intention was scientifically appropriate, namely to gain further insight about IHCAs through a systematic review. In the other three categories, fault can be found:

- The errors in the coding of data should not have been made. The coauthors Nazareth and Tai, who are credited with doing the coding, have good enough credentials to not lay the blame on lack of experience: Nazareth is a Professor at UCL and is Scientific Director of the British Medical Research Council's General Practice and Research Framework, and Tai has coauthored several articles over the past two decades that appeared in refereed medical journals. An explanation for the miscoding in terms of experience of the coders is not apparent.
- The design of the study has been criticized as lumping together studies which are too heterogeneous in their design, interventions, and outcomes [8,9]. The protocol might have been more rigorously vetted by the Cochrane Review Group, and the authors should have been more cautious in their interpretation of results and emphasized the weakness of the design in their publicity.
- The reporting of the work suggests possible bias. The authors and their employers have sensationalized a result that catches the media's attention. For some observers, the review appeared biased in that the authors, who are affiliated with medical institutions, concluded that patients should listen to their doctor, instead of seeking help on the Internet.

Purchase [24] claims that a partial solution to this first-order problem is the institutionalization of quality controls. In the 1970s, good laboratory practice regulations were introduced, but comparable regulations do not exist for meta-analyses. For quality control of a meta-analysis the scientific community relies on the research team, the researchers' institution, and the referees. A medical research institution, such as the UCL Medical School, presumably embraces results from its researchers that can earn mass media coverage and is not the appropriate institution to prevent sensationalizing. Referees can not be expected to detect when laboratory data are intentionally modified [25]; however, in this case they could have been expected to detect when data available to them are miscoded. Problems with refereeing have been frequently noted and in particular for the Cochrane Database [26].

Open commentary, as exists for the Cochrane Database after a publication, is one way to identify flaws. Extending the open commentary to the refereeing phase might reduce the likelihood of something going to press with errors. A submitted article might be available to the public and a community of hundreds of registered scientists could be invited to make anonymous comment. Submissions online would require extensive online commenting that reached a consensus before a submission could be considered “published.” Other approaches to increase the commentary on the research process include refereeing the protocol phase [27], which is done by the Cochrane Collaboration but apparently not with the necessary rigor or topic expertise.

### Second-Order Problem

The second-order problem is a press release and subsequent mass media coverage of the release. Winsten's classic study of science and the media shows how the truth is repeatedly misrepresented by journalists and researchers: “The most striking finding which emerged from the interviews [of medical journalists] is the dominant distorting influence of the competitive force in journalism.… As economic competition among hospitals has intensified, they have begun to compete aggressively for publicity.… With increasing frequency…scientists…are using the media to attach their names to important findings before their competitors do.… The result has been a spiraling competition, sometimes characterized by exaggerated claims” [28].

Online media have stimulated further competition [29]. The case of the IHCA review reflects these pressures. The UCL press release contained inaccuracies, even if the review would have been scientifically sound, in order to gain mass media attention. For instance, the subtitle of the press release was “Knowledge may be hazardous to web consumers' health.” In truth, the IHCA review was not about Web applications, per se, but about IHCAs, which are defined more broadly than “Web applications.” However, writing a news article about IHCAs is less likely to catch attention than an article about the Web. The UCL press release did not introduce and define the term IHCA, and Murray issued statements that implied the Web was the issue. By the time the information from the press release made it into the mass media, the material had been modified enough to lose any mention of IHCAs. For instance, the *Time* article said, “People who use the Web to get information about their chronic diseases often wind up in worse shape than before they logged on.”

One way for researchers to prevent the mass media from misrepresenting the truth is for researchers to understand how the media work and to interact with the media accordingly [28]. Murray should have known that her words might be twisted to emphasize what would sell newspaper space and should not have wildly speculated. The reputations of the Cochrane Collaboration and UCL partially account for the wide dissemination of the original press release. Yet, neither organization has taken adequate steps to undo the impact of the media reporting on the IHCA review.

The honesty of the press could be improved with the Internet [30]. Online health care mass media publications could allow the public to make comments on news articles. Rating techniques, such as employed at eBay and Slashdot, might be used to give prominence to quality feedback [31].

### Third-Order Problem

The third-order problem concerns the long-term impact of the mass media. While electronic publications might be erased from a computer or marked as retracted, this does not consistently happen. Furthermore, some of the mass media coverage of the IHCA review is on paper and sits on people's bedside tables with no practical way to be retracted [32].

Although this author did not (yet) find any citations to the IHCA review in Web of Science, previous studies have confirmed that a retracted scientific publication may continue to have impact without readers recognizing its retracted status. For instance, one study [33] tracking the citation pattern of 82 retracted articles revealed that, together, they were cited 733 times after their retraction, but only a small fraction of the citations referred to the retraction. In the case of the mass media, retracted publications might be read by people without them seeing the separate retraction notice.

If and when the revised IHCA review is published, what could it say that would undo the effect of the original publication? If the conclusion is that IHCAs result in improved clinical outcomes, then the medical profession will want to closely study the protocol and might have grounds to discredit the conclusion. The media trumpeted the IHCA review conclusion partly because it was counterintuitive but was backed by top-notch institutions. If the conclusion becomes intuitive, then the media are unlikely to be interested in it.

The reactions to the IHCA review in patient online discussions highlight the importance of virtual communities in helping patients deal with published information. Simple extensions to Web-based, patient, discussion systems could help patients connect to Web-based publications. For instance, when a patient posts a message to a Web-based discussion board, the Web system could parse the message and provide links from the message to relevant articles on the Web. Patients might follow the links and engage in discourse about the validity and implications of the literature. This might lessen the potential ill effects of publications that are wrong or misleading.

### Conclusions

This special medical publishing event was marked by incorrect coding and a desire for maximum publicity. The IHCA review authors, their employers, and the Cochrane Collaboration were responsible for quality control, and failed. The mass media played their part by widely publicizing a sensational message but not reacting to the notice that that sensational message was false. The false result that patients are clinically harmed by interactive applications was very strongly delivered to patients worldwide. The broad lesson to be re-learned is that potentially sensational results should be carefully scrutinized before being sensationalized.

## Abbreviations

IHCA: interactive health communication applications
UCL: University College London
